# Supplementary material for: Cumulative incidence, prevalence, seroconversion, and associated factors for SARS-CoV-2 infection among healthcare workers of a University Hospital in Bogotá, Colombia
Source: PLoS One. 2022 Sep 19;17(9):e0274484. doi: 10.1371/journal.pone.0274484 (PMC9484677; doi:10.1371/journal.pone.0274484)
Supplement: S1 Methods — (DOCX) [file pone.0274484.s001.docx]

1. **Supplementary Laboratory Methods**

Sampling and laboratory methods:

At the clinical laboratory of Hospital Universitario San Ignacio (HUSI), participants signed an electronic informed consent, and donated 7 ml of venous blood. Serum was obtained by centrifugation.

RBD Hemaglutination assay (HA)

The assay was performed as described recently by Townsend et al [(Townsend et al. 2021)](https://paperpile.com/c/I88CGM/G5w1). Reagents to perform (IH4-RBD) and standardize the assay (monoclonal antibodies CR3022 and EY6A) were provided by Dr. Townsend. Venous O− blood (to avoid agglutination by natural agglutinins in the plasma) was obtained in K EDTA containing tubes washed three times with PBS, and red blood cells (RBC) were diluted to a final concentration of 2%. Plasmas were diluted 1:40 in 50 μL, then 50 μL of 2% RBC were added, followed by addition of 100 ng of the IH4-RBD in 50 μL PBS or 50 μL PBS as negative control. After 1 h incubation, plates were tilted for ~30 s, photographed, and read by two investigators separately. Agglutination was determined as complete loss of teardrop formation by the red cells, any formation of a teardrop was regarded as negative.

To validate the HA we tested 181 serum samples (40 samples positive and 140 negative samples) from our previous project [(Ariza et al. 2021)](https://paperpile.com/c/I88CGM/DnRI). Concordance with Abbott IgG CLIA was good (Cohen´s Kappa 0.917 95% CI 0.8366-0.9974). To further validate the assay, we tested two monoclonal antibodies (CR3022 and EY6A) that recognize cross-reactive epitopes and panel 20/118 (that has serums with varying levels of antibodies against SARS-CoV-2) and serum 20/130 provided by WHO, with expected results (Supplementary figure 1). Serum 20/130 that gave a titer of 1/5120 in HA (Supplementary Figure 1) has been calibrated against the WHO international standard and has 1300 IU/mL (95% confidence limits 981-1719) for neutralizing antibody activity and 502 (95% confidence limits 382-660) Anti-receptor-binding domain IgG BAU/mL [(Knezevic et al. 2021)](https://paperpile.com/c/I88CGM/Gogr). Similar results with the WHO standard 20/130 serum have been reported recently (Ertesvåg et al. 2022).

Chemiluminescent assay (CLIA)

SARS-CoV-2 IgG tests (Abbott Colombia) that recognize the viral nucleoprotein were performed on an Abbott Architect i1000 analyzer, following the manufacturer’s protocol, and as previously described [(Ariza et al. 2021)](https://paperpile.com/c/I88CGM/DnRI). A single lot of positive and negative controls were run at the start of each batch of antibody testing. Samples with a signal-to-cutoff (S/CO) ratio greater than or equal to 1.4 were considered positive.

Enzyme linked fluorescence assay (ELFA)

The VIDAS Anti-SARS CoV-2 IgM two-step sandwich ELFA that recognizes the viral Spike protein was performed on a VIDAS analyzer (BioMérieux, Marcy-l’Etoile, France) and as previously described [(Ariza et al. 2021)](https://paperpile.com/c/I88CGM/DnRI). An index is calculated as the ratio between the relative fluorescence value measured in the sample and the relative fluorescence obtained for a calibrator (humanized recombinant anti-SARS CoV-2 IgM) and interpreted as negative (index<1) or positive (index≥1) (20).

RBD Enzyme-linked immunosorbent assay (ELISA)

IgG antibodies specific for the Wuhan RBD were assessed as previously described with minor modifications [(Stadlbauer et al. 2020)](https://paperpile.com/c/I88CGM/z6rN): 96-well vinyl microtiter ELISA plates (Thermo Electron Corporation, Milford, MA) were coated with either 1ug/well RBD (Purified His-tagged produced in 293 cells from Atum Biologicals Newark, CA) or PBS (negative control) and incubated overnight at 4C. After blocking, a 1/40 dilution of plasma samples were deposited in each well. After incubation, the following sequence of reagents was added: biotin-labeled goat anti- human IgG (Kirkegaard & Perry Laboratories [KPL], Gaithersburg, MD); streptavidin-peroxidase (KPL) and tetramethyl benzidine substrate (KPL). Plasma samples were used as negative and positive controls. Samples were considered positive if the optical density in the experimental wells was 0.1 units and two-fold greater than the optical density in the corresponding negative control wells. Control plasmas had comparable DO from plate to plate. Titration in the ELISA of selected serums from panel 20/118 and serum 20/130 provided by WHO gave expected results.

RT-PCR

RT-PCR was performed in accredited HUSI clinical laboratory on nasopharyngeal swab or aspirates using the VIASURE™ Real-Time PCR Detection Kit plates (CerTest BIOTEC, Zaragoza, Spain). Detection is performed in a one-step real-time RT format, where reverse transcription and amplification of the specific target sequence occurs in the same reaction well. The isolated RNA target is transcribed to generate complementary DNA by reverse transcriptase, followed by amplification of a ORF1ab and N gene region for SARS-CoV-2 using specific primers and a fluorescently labeled probe.

**References**

[Ariza, Beatriz, Ximena Torres, Diana Salgado, Magda Cepeda, Carlos Gómez Restrepo, Julio Cesar Castellanos, Fernando Suárez-Obando, et al. 2021. “Seroprevalence and Seroconversion Rates to SARS-CoV-2 in Interns, Residents, and Medical Doctors in a University Hospital in Bogotá, Colombia.” *Infectio: Revista de la Asociacion Colombiana de Infectologia* 25 (3): 145.](http://paperpile.com/b/I88CGM/DnRI)

Ertesvåg, Nina Urke, Julie Xiao, Fan Zhou, Sonja Ljostveit, Helene Sandnes, Sarah Lartey, Marianne Sævik, et al. 2022. “A Rapid Antibody Screening Haemagglutination Test for Predicting Immunity to SARS-CoV-2 Variants of Concern.” *Communications Medicine* 2 (1): 1–11.

[Knezevic, Ivana, Giada Mattiuzzo, Mark Page, Philip Minor, Elwyn Griffiths, Micha Nuebling, and Vasee Moorthy. 2021. “WHO International Standard for Evaluation of the Antibody Response to COVID-19 Vaccines: Call for Urgent Action by the Scientific Community.” *The Lancet. Microbe*, October. https://doi.org/](http://paperpile.com/b/I88CGM/Gogr)[10.1016/S2666-5247(21)00266-4](http://dx.doi.org/10.1016/S2666-5247(21)00266-4)[.](http://paperpile.com/b/I88CGM/Gogr)

[Stadlbauer, Daniel, Fatima Amanat, Veronika Chromikova, Kaijun Jiang, Shirin Strohmeier, Guha Asthagiri Arunkumar, Jessica Tan, et al. 2020. “A Detailed Protocol for a Serological Assay to Detect SARS-CoV-2 Seroconversion in Humans: Antigen Production and Test Setup.” *Current Protocols in Microbiology* 57 (1): 1–13.](http://paperpile.com/b/I88CGM/z6rN)

[Townsend, Alain, Pramila Rijal, Julie Xiao, Tiong Kit Tan, Kuan-Ying A. Huang, Lisa Schimanski, Jiandong Huo, et al. 2021. “A Haemagglutination Test for Rapid Detection of Antibodies to SARS-CoV-2.” *Nature Communications* 12 (1): 1951.](http://paperpile.com/b/I88CGM/G5w1)
