# Supplementary figures and images for: Cumulative incidence, prevalence, seroconversion, and associated factors for SARS-CoV-2 infection among healthcare workers of a University Hospital in Bogotá, Colombia
Source: PLoS One. 2022 Sep 19;17(9):e0274484. doi: 10.1371/journal.pone.0274484 (PMC9484677; doi:10.1371/journal.pone.0274484)

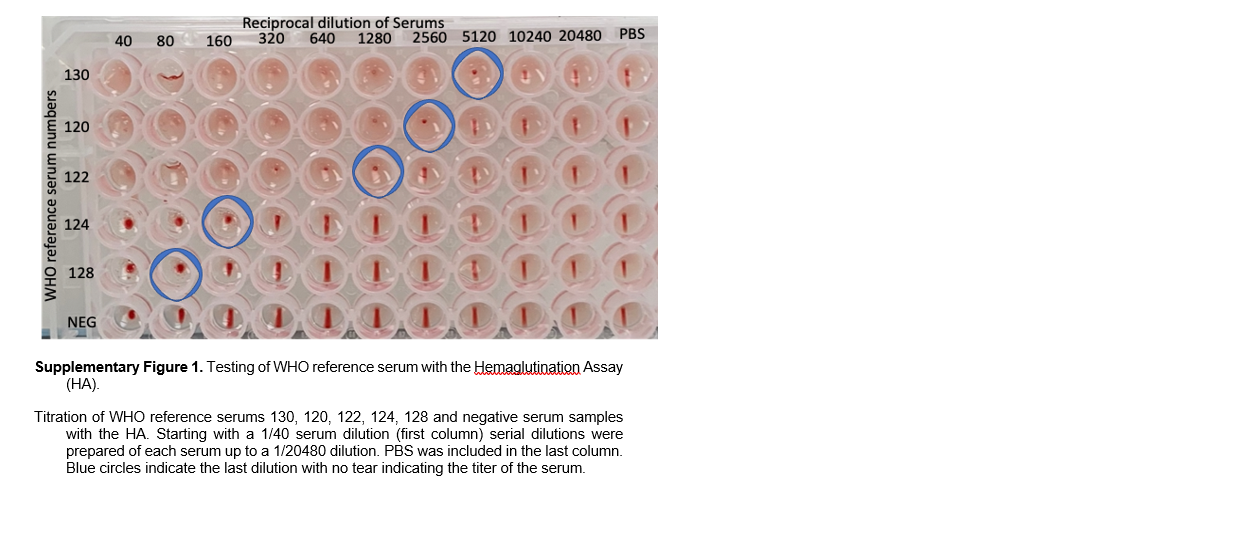

Supplement: S1 Fig — Titration of WHO reference serums 130, 120, 122, 124, 128, and negative serum samples with the HA. Starting with a 1/40 serum dilution (first column) serial dilutions were prepared of each serum up to a 1/20480 dilution. PBS was included in the last column. Blue circles indicate the last dilution with no tear indicating the titer of the serum. (TIF) [file pone.0274484.s003.tif]

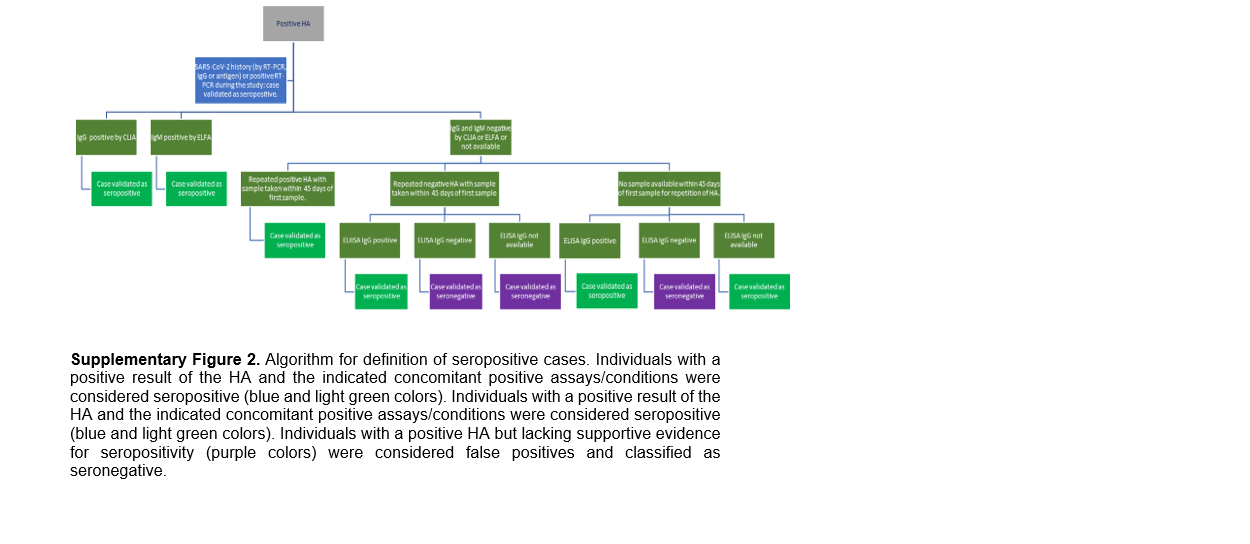

Supplement: S2 Fig — Individuals with a positive result of the HA and the indicated concomitant positive assays/conditions were considered seropositive (blue and light green colors). Individuals with a positive HA, but lacking supportive evidence for seropositivity (purple colors) were considered false positives and classified as seronegative. (TIF) [file pone.0274484.s004.tif]

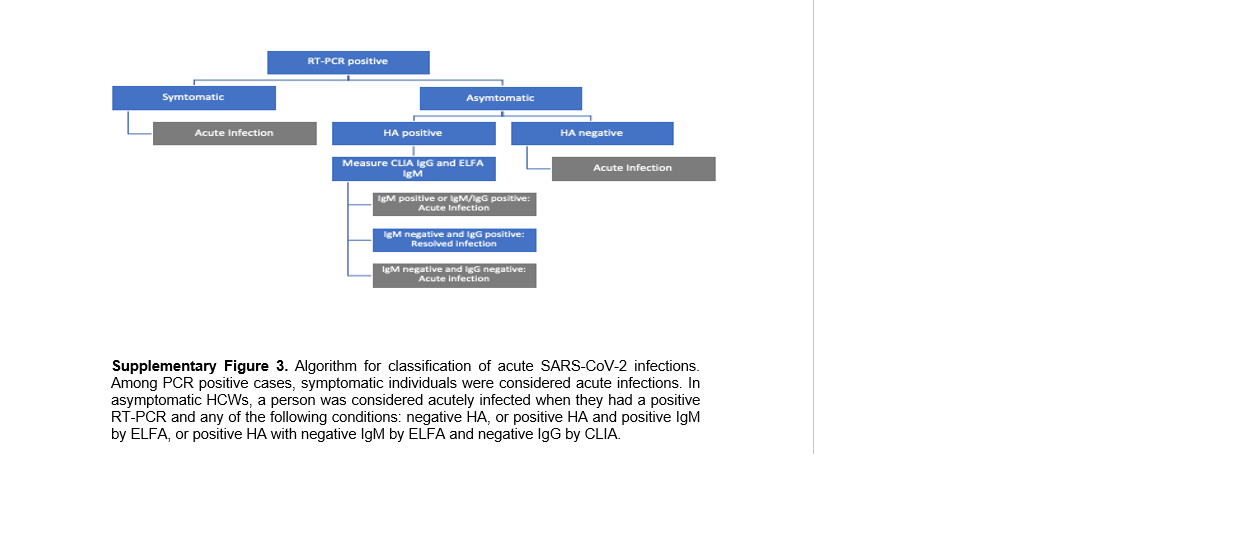

Supplement: S3 Fig — Among PCR positive cases, symptomatic individuals were considered acute infections. In asymptomatic HCWs, a person was considered acutely infected when they had a positive RT-PCR and any of the following conditions: negative HA, or positive HA and positive IgM by ELFA, or positive HA with negative IgM by ELFA and negative IgG by CLIA. (TIF) [file pone.0274484.s005.tif]
