# Supplementary material for: Cumulative incidence, prevalence, seroconversion, and associated factors for SARS-CoV-2 infection among healthcare workers of a University Hospital in Bogotá, Colombia
Source: PLoS One. 2022 Sep 19;17(9):e0274484. doi: 10.1371/journal.pone.0274484 (PMC9484677; doi:10.1371/journal.pone.0274484)
Supplement: S1 Table — November 2020. 1Column-based percentages. (DOCX) [file pone.0274484.s006.docx]

**Supplementary Table 1.** Comparison of healthcare workers (HCWs) at Hospital Universitario San Ignacio who participated and did not participate in the study. November 2020.

| **Participant Characteristics** | **HCWs who did not participate**  **(n = 685)**  **n (%)^1^** | **HCWs who participate**  **(n = 259)**  **n (%)^1^** |
| --- | --- | --- |
| Sex (n, %) |  |  |
| Female | 493 (72.0%) | 1940 (74.7%) |
| Male | 192 (28.0%) | 657 (25.3%) |
| Age (years), (median, IQR) | 33.0 (26.0 - 40.0) | 34.2 (28.3 - 41.6) |
| Age (years), (n, %) |  |  |
| < 35 | 384 (56.1%) | 1377 (53.0%) |
| 35 - 44 | 189 (27.6%) | 772 (29.7%) |
| ≥ 45 | 112 (16.4%) | 448 (17.3%) |
| Type of occupation (n, %) |  |  |
| Direct patient care | 521 (76.0%) | 2026 (78.0%) |
| Administrative staff | 164 (24.0%) | 571 (22.0%) |

^1^Column-based percentages
