# Supplementary material for: Cumulative incidence, prevalence, seroconversion, and associated factors for SARS-CoV-2 infection among healthcare workers of a University Hospital in Bogotá, Colombia
Source: PLoS One. 2022 Sep 19;17(9):e0274484. doi: 10.1371/journal.pone.0274484 (PMC9484677; doi:10.1371/journal.pone.0274484)
Supplement: S2 Table — 1Column-based percentages. 2ICU = Intensive Care Unit. 3HCW who has worked in the COVID area sometime since March 2020. 4HCW who was less than 6 feet away from an infected person (laboratory-confirmed or a clinical diagnosis) for a cumulative total of 15 minutes without personal protection elements sometime since March 2020. 5Shared transportation was defined as the use of any public or collective transport. 6History of smoking in the last year. 7History of influenza vaccination in the last year. 8Self-reported pre-existing medical condition. (DOCX) [file pone.0274484.s007.docx]

**Supplementary Table 2.** Comparison of healthcare workers at Hospital Universitario San Ignacio who returned and did not return for the follow-up in the prospective cohort for studying seroconversion (December 15, 2020 to February 26, 2021).

| **Participant Characteristics** | **Returned (had two samples) (n= 1654)**  **n (%)^1^** | **Did not return (baseline sample only) (n= 943)**  **n (%)^1^** |
| --- | --- | --- |
| Sex (n, %) |  |  |
| Female | 1307 (79.0) | 633 (67.1) |
| Male | 347 (21.0) | 310 (32.9) |
| Age (years) (n, %) |  |  |
| < 35 | 736 (44.5) | 641 (68.0) |
| 35 - 44 | 569 (34.4) | 203 (21.5) |
| ≥ 45 | 349 (24.7) | 99 (10.5) |
| Type of occupation (n, %) |  |  |
| Direct patient care | 1246 (75.3) | 777 (82.4) |
| Administrative | 408 (24.7) | 166(17.6) |
| Type of direct patient care worker (n, %) |  |  |
| Administrative | 408 (24.7) | 163 (17.3) |
| Physician | 276 (16.7) | 342 (36.3) |
| Nurse | 682 (41.2) | 318 (33.7) |
| Other | 288 (17.4) | 120 (12.7) |
| Main Service (n, %) |  |  |
| Administrative office | 267 (16.2) | 106 (11.4) |
| Emergency room | 218 (13.2) | 174 (18.6) |
| General wards | 436 (26.4) | 275 (29.5) |
| ICU^2^ | 179 (10.8) | 87 (9.3) |
| Surgical areas | 170 (10.3) | 115 (12.3) |
| Ambulatory and diagnostic services | 382 (23.1) | 176 (18.9) |
| Type of respiratory protection (n, %) |  |  |
| Cloth mask | 63 (3.9) | 37 (4.1) |
| Surgical mask | 712 (43.7) | 294 (32.4) |
| N-95 respirator | 854 (52.4) | 576 (63.5) |
| History of close contact^3^ (n, %) |  |  |
| Yes | 762 (46.7) | 457 (51.2) |
| No | 869 (53.3) | 435 (48.8) |
| Type of work (n, %) |  |  |
| Remote-work | 198 (12.1) | 106 (11.6) |
| Non remote work | 1433 (87.9) | 811 (88.4) |
| COVID work^4^ (n, %) |  |  |
| Yes | 812 (49.7) | 530 (58.1) |
| No | 512 (56.4) | 383 (41.9) |
| Shift (n, %) |  |  |
| Day shift | 1127 (69.1) | 563 (61.4) |
| Night shift | 504 (30.9) | 355 (38.7) |
| Type of transportation^5^ (n, %) |  |  |
| Unshared | 750 (46.1) | 485 (54.0) |
| Shared | 878 (53.9) | 413 (46.0) |
| Work in more than one institution (n, %) |  |  |
| Work at only one institution | 1495 (91.9) | 806 (88.2) |
| Work at two or more institutions | 131 (8.1) | 108 (11.8) |
| Smoking in the previous year^6^ (n, %) |  |  |
| Yes | 203 (12.5) | 136 (12.8) |
| No | 1424 (87.5) | 749 (87.2) |
| Influenza vaccination in the previous year^7^ (n, %) |  |  |
| Yes | 691 (42.7) | 283 (30.3) |
| No | 927(57.3) | 581 (62.3) |
| Body Max Index (kg/m^2^) (n, %) |  |  |
| Low and normal (<25) | 953 (58.5) | 550 (62.1) |
| Overweight (25 – 29-9) | 544 (33.4) | 282 (31.8) |
| Obesity (>30) | 131 (8.1) | 54 (6.1) |
| Comorbidities^8^ (n, %) |  |  |
| Any comorbidity | 306 (18.8) | 121 (13.5) |
| Without comorbidity | 1325 (81.2) | 773 (86.5) |

^1^Column-based percentages. ^2^ICU= Intensive Care Unit. ^3^HCW who has worked in the COVID area sometime since March 2020. ^4^HCW who was less than 6 feet away from an infected person (laboratory-confirmed or a clinical diagnosis) for a cumulative total of 15 minutes without personal protection elements sometime since March 2020. ^5^Shared transportation was defined as the use of any public or collective transport. ^6^History of smoking in the last year. ^7^History of influenza vaccination in the last year. ^8^Self-reported pre-existing medical condition.
