# Supplementary material for: Cumulative incidence, prevalence, seroconversion, and associated factors for SARS-CoV-2 infection among healthcare workers of a University Hospital in Bogotá, Colombia
Source: PLoS One. 2022 Sep 19;17(9):e0274484. doi: 10.1371/journal.pone.0274484 (PMC9484677; doi:10.1371/journal.pone.0274484)
Supplement: Attachment — Submitted filename: Response to reviewers_15-7-22.docx [file pone.0274484.s008.docx]

July 15, 2022

**Reviewer and Academic Editor**

**PLOS ONE**

Dear reviewer and academic editor

We want to thank the reviewer for his insightful and helpful comments. A detailed response to each and every comment is provided below in bold. In addition, please find a reply to each and every one of the “Journal requirements” also in bold.

**Journal Requirements:**

1. Please ensure that your manuscript meets PLOS ONE’s style requirements, including those for file naming.

**Answer: The manuscript and files have been adjusted following the style requirements.**

2. Please state what role the funders took in the study.

**Answer: The following statement is added in Financial Disclosure: The funders had no role in study design, data collection and analysis, decision to publish, or preparation of the manuscript. We added the financial disclosure statement in the cover letter as advised.**

3. Thank you for stating the following in the Acknowledgments Section of your manuscript:

"This study was funded by Pontificia Universidad Javeriana, Hospital Universitario San Ignacio, and Fundación Bolívar Davivienda."

We note that you have provided funding information that is not currently declared in your Funding Statement. However, funding information should not appear in the Acknowledgments section or other areas of your manuscript. We will only publish funding information present in the Funding Statement section of the online submission form.
Please remove any funding-related text from the manuscript and let us know how you would like to update your Funding Statement. Currently, your Funding Statement reads as follows:

"This study was funded by Pontificia Universidad Javeriana, Hospital Universitario San Ignacio, and Fundación Bolívar Davivienda.

We are grateful to Dr Tiong Kit Tan and Prof Alain Townsend for technical discussion and for supplying the HAT reagents for this study, and to the donors of the Townsend-Jeantet Prize Charitable Trust Charity No 1011770 for support."

Please include your amended statements within your cover letter; we will change the online submission form on your behalf.

**Answer: We added the following statement of financial disclosure in the cover letter: “This study was funded by Pontificia Universidad Javeriana, Hospital Universitario San Ignacio, and Fundación Bolívar Davivienda. The funders had no role in study design, data collection and analysis, decision to publish, or preparation of the manuscript.”**

4. Please complete your Competing Interests on the online submission form to state any Competing Interests.

**Answer: The following statement was added in the Competing Interests section: The authors have declared that no competing interests exist. (Page 36 Line 544).**

5. In your Data Availability statement, you have not specified where the minimal data set underlying the results described in your manuscript can be found. PLOS defines a study's minimal data set as the underlying data used to reach the conclusions drawn in the manuscript and any additional data required to replicate the reported study findings in their entirety. All PLOS journals require that the minimal data set be made fully available.

Upon re-submitting your revised manuscript, please upload your study’s minimal underlying data set as either Supporting Information files or to a stable, public repository and include the relevant URLs, DOIs, or accession numbers within your revised cover letter. For a list of acceptable repositories, please see http://journals.plos.org/plosone/s/data-availability#loc-recommended-repositories. Any potentially identifying patient information must be fully anonymized.

Important: If there are ethical or legal restrictions to sharing your data publicly, please explain these restrictions in detail. Please see our guidelines for more information on what we consider unacceptable restrictions to publicly sharing data: http://journals.plos.org/plosone/s/data-availability#loc-unacceptable-data-access-restrictions. Note that it is not acceptable for the authors to be the sole named individuals responsible for ensuring data access.

We will update your Data Availability statement to reflect the information you provide in your cover letter.

**Answer: We uploaded to the editorial manager as supporting information an Excel file with the minimal dataset of our study and the data dictionary. The data are free of personal identifiers. We cited as S2 minimal dataset in the supporting information section of the manuscript. We also provided this information in the cover letter.**

**Reply to the Reviewer´s Comments to the Author:**

1. Is the manuscript technically sound, and do the data support the conclusions?

The manuscript must describe a technically sound piece of scientific research with data that supports the conclusions. Experiments must have been conducted rigorously, with appropriate controls, replication, and sample sizes. The conclusions must be drawn appropriately based on the data presented.

Reviewer #1: Partly

2. Has the statistical analysis been performed appropriately and rigorously?

Reviewer #1: Yes

3. Have the authors made all data underlying the findings in their manuscript fully available?

The PLOS Data policy requires authors to make all data underlying the findings described in their manuscript fully available without restriction, with rare exception (please refer to the Data Availability Statement in the manuscript PDF file). The data should be provided as part of the manuscript or its supporting information, or deposited to a public repository. For example, in addition to summary statistics, the data points behind means, medians and variance measures should be available. If there are restrictions on publicly sharing data—e.g. participant privacy or use of data from a third party—those must be specified.

Reviewer #1: Yes

4. Is the manuscript presented in an intelligible fashion and written in standard English?

PLOS ONE does not copyedit accepted manuscripts, so the language in submitted articles must be clear, correct, and unambiguous. Any typographical or grammatical errors should be corrected at revision, so please note any specific errors here.

Reviewer #1: Yes

My main concerns are:

1. The authors present a legit conclusion, but the manuscript would benefit from a clear hypothesis. Particularly the authors are advised to address, what is the prevalence, incidence and likely epidemiological role of asymptomatic – either pre-symptomatic or asymptomatic – SARS-CoV-2 infections, particularly if nosocomially acquired, among the healthcare personnel in the University Hospital in Bogota. These results could be further compared to healthcare workers eg. in Sweden where very limited preventative measures were implemented. See Pimenoff et al. 2021 PLoS One. doi:10.1371/journal.pone.0260453.

**Answer: Our objectives were to determine SARS-CoV-2 infection cumulative incidence, prevalence, seroconversion, and associated factors among** **healthcare workers (HCWs) of a University Hospital in Bogota, Colombia and we constructed a model in which we evaluated different sociodemographic, clinical, and occupational characteristics and their association with the presence of SARS-CoV-2 infection.**

**Furthermore, in a random subsample of HWCs in which RT-PCR was carried out to assess acute infection, we found a low prevalence (2.8%). Asymptomatic infections accounted for half of them (55%), highlighting the importance of screening as a containment measure in healthcare settings.**

**In the discussion (Pages 30-31 Line 408 to 412) we now reference for comparison the study suggested by the reviewer, elaborating on the prevalence, incidence, and likely epidemiological role of asymptomatic – either pre-symptomatic or asymptomatic – SARS-CoV-2 infections, among healthcare personnel.**

**However, we did not design the study to evaluate the role of asymptomatic and presymptomatic infections in the occurrence of new infections in our cohort as was done in 1. Pimenoff VN, Elfström M, Lundgren KC, Klevebro S, Melen E, Dillner J. Potential SARS-CoV-2 infectiousness among asymptomatic healthcare workers. PLoS One. 2021;16(12 December):1–7, 2. For this reason, we prefer not to formulate a hypothesis similar to what is presented in the cited paper.**

2. It is surprising to see weight associate to the risk of acquiring SARS-CoV-2 infection. However, this is likely a bias in the data as overweight is one of the risk factors for severe COVID-19 and thus overweight HCWs may be more willing to participate in SARS-CoV-2 infection screening than non-risk category HCWs. A systematic sensitivity analysis should be performed to identify if the dataset presented in this study is biased by selection of any severe COVID-19 disease related risk factors.

**Answer: Since we did not include all of the target population in the study, we cannot exclude a selection bias related to being overweight or having any other associated disease or factor. However, the differential participation of individuals at higher risk of infection or severe disease (since the characteristic should also be a risk factor for infection to generate bias) could generate an overestimation of the prevalence and incidence of infection (as stated in the limitations of the study), but not necessarily an overestimation of the association between obesity and the risk of infection, as long as the contrast between the individuals in categories defined by weight be valid, that is, unconfounded. This is achieved if the categories have the same distribution of all other risk factors for infection. In our analytical model, we adjusted for the known or hypothesized risk factors that could be measured at the time of the study; however, some uncontrolled confounding may persist and explain part of the associations observed. To explain this, we included the previous sentence in the limitations of the study (Page 27 Line 423 to 426).**

**In addition, our conclusion is strengthened by the fact that we found a gradient in the risk of SARS-CoV-2 infection according to weight. Finally, a recent publication from a South African study found an independent association between obesity and overweight and acquiring SARS-CoV-2 infection (Reference 40 of the new version of the paper). To support this, we included the following sentence (Page 32-33 Line 457 to 460) “A study in South Africa found that overweight and obesity in HCWs were associated with SARS-CoV-2 infection in the multivariate analysis [40]”. (Stead D, Adeniyi OV, Singata-Madliki M, Abrahams S, Batting J, Jelliman E, et Al. Cumulative incidence of SARS-CoV-2 and associated risk factors among healthcare workers: a cross-sectional study in the Eastern Cape, South Africa. BMJ Open. 2022 Mar 18;12(3):e058761. doi: 10.1136/bmjopen-2021-058761.)**

**To address the possible overestimation of SARS-CoV-2 infection prevalence and incidence due to potential differential participation of obese/overweight HCWs in our study, we obtained from the Human Resources Office of HUSI the global information on comorbidities of its HCWs, which is estimated based on a convenience sample different to the one we used (see table below). The prevalence of overweight was 34.7%, and obesity was 11.3% in 2020. In our study, we found frequencies of overweight (n= 826; 32.89%) and obese (n= 185; 7.4%) HCWs lower than the estimated for the overall population of the hospital. Thus, it is unlikely that we have overestimated the SARS-CoV-2 prevalence and incidence due to this differential participation**.

**Finally, the prevalences of other clinically relevant factors in the HUSI HCWs were: hypothyroidism 4.0%, arterial hypertension 2.3% and smoking 9.8%. In contrast, in our study the corresponding prevalences were 4.4%, 5.1% and 12.6%. Despite a slight overrepresentation of HCWs with arterial hypertension and smoking in our study, neither of them was significantly associated to an increased risk of acquiring SARS-Cov-2 infection.**

| **Severity Risk Factor** | **Global estimated HUSI (%)** | **Our Study (%)** |
| --- | --- | --- |
| **Overweight** | **34.7** | **32.9** |
| **Obesity** | **11.3** | **7.4** |
| **Hypothyroidism** | **4.0** | **4.4** |
| **Arterial Hypertension** | **2.3** | **5.1** |
| **Smoking** | **9.8** | **12.6** |

3. A figure of the prevalence data as a function of time would be a good way to visualize the data and related it to waves of the pandemic and the dominant variant.

**Answer: The figure proposed by the reviewer is cited in the manuscript (Page18 Line 288-291) as well as the figure caption (Page 23 Line 309-323). This figure will be uploaded as a separate file meeting the requirements.**

**Fig 1. Seroprevalence and cumulative incidence of SARS-CoV-2 infection in Hospital Universitario San Ignacio (HUSI) healthcare workers (HCWs), and Bogota's COVID-19 epidemic curve.** A. The SARS-CoV-2 seroprevalence in HUSI HCWs was 21.5% between November 17, 2020 and February 12, 2021 (n = 2,597) and 24.8% (n = 1,654) between December 15, 2020 and February 26, 2021. B. The SARS-CoV-2 epidemic curve of Bogota between March 2020 and February 2021 shows two epidemic waves. The first one began in June 2020 and ended approximately in October 2020, and the second one began in November 2020 and ended in February 2021. In this last epidemic wave, Gamma (P.1) and Mu (B.1.621) variants were introduced in the city. C. The SARS-CoV-2 cumulative incidence in HUSI HCWs was 35.7% (927/2,597) between March 6, 2020 and February 12, 2021. *The numbers of SARS-CoV-2 infection cases were taken from: https://saludata.saludcapital.gov.co/osb/index.php/datos-de-salud/enfermedades-trasmisibles/covid19/

4. Manuscript text could have a bit more flow, revision of the text is advised.

**Answer: The suggested revision was carried out by an English editing service. We uploaded the revision certificate provided by this service.**
